# Endotoxin and Kupffer Cell Activation in Alcoholic Liver Disease

**Published:** 2003

**Authors:** Michael D. Wheeler

**Affiliations:** Michael D. Wheeler, Ph.D., is an assistant professor in the Center for Alcohol Studies at the University of North Carolina, Chapel Hill

**Keywords:** alcoholic liver disorder, chronic AODE (alcohol and other drug effects), endotoxins, Kupffer cell, cell growth and differentiation, biological activation, cell signaling, cytokines, intestinal cell, epithelium, membrane permeability, treatment method

## Abstract

One central component in the complex network of processes leading to the development of alcoholic liver disease is the activation of immune cells residing in the liver (i.e., Kupffer cells) by a substance called endotoxin, which is released by bacteria living in the intestine. Alcohol consumption can lead to increased endotoxin levels in the blood and liver. When activated, Kupffer cells produce signaling molecules (i.e., cytokines) that promote inflammatory reactions as well as molecules called reactive oxygen species (ROS), which can damage liver cells. Endotoxin activates Kupffer cells by interacting with a complex of protein molecules that are located on the outside of the Kupffer cell or which extend into the cell. Binding of endotoxin alters the activities of the proteins in this complex so that they trigger a cascade of biochemical signals in the Kupffer cell, resulting in cytokine and ROS production and, ultimately, liver damage. Because alcohol can enhance endotoxin release and, therefore, Kupffer cell activation, novel approaches to inhibit these processes might help prevent or ameliorate alcoholic liver disease.

Alcoholic liver disease progresses through several stages of tissue damage and liver dysfunction. One of the early stages, alcohol-induced steatohepatitis, is characterized by the accumulation of fat molecules in the liver tissue, accompanied by the migration into the liver of cells associated with inflammation processes. These inflammation-promoting cells are attracted to the liver largely because of the activities of a type of immune cell called Kupffer cells, which reside there.^1^ To investigate and clarify the contribution of Kupffer cells to the development of alcohol-induced liver injury, researchers studied rats in which they could induce liver disease by continuously administering alcohol. If the animals were first treated with a chemical (i.e., gadolinium chloride) that specifically destroyed their Kupffer cells, they did not develop alcohol-induced liver injury (Adachi et al. 1994). This key finding supports the idea that Kupffer cells play an important role in alcohol’s detrimental effects on liver function.

Alcohol activates Kupffer cells primarily through the action of a substance called endotoxin, which is released by certain bacteria normally present in the intestine of humans and other animals. This article describes the interaction between endotoxin and Kupffer cells, particularly the mechanisms by which endotoxin activates signaling pathways within the Kupffer cells, and how these mechanisms contribute to liver injury. The article concludes by discussing how these findings could be translated into new approaches to treating alcoholic liver disease.

## What Are Kupffer Cells?

Kupffer cells belong to a class of immune cells called macrophages, which are found throughout most tissues. Macrophages take various actions to eliminate foreign materials (e.g., bacteria or bacterial products) from the body. For example, macrophages can ingest and destroy foreign materials or secrete immune molecules as well as molecules that regulate the functions of other cells involved in the immune response. These regulatory molecules are called cytokines.

Kupffer cells are macrophages that reside in the liver. Their main role— removing bacteria and foreign proteins from the blood—is essential to the liver’s primary function, which is cleansing the blood of foreign materials and toxic substances. When no foreign materials are present, Kupffer cells are in a resting state. They can be activated by numerous molecules, including bacterial endotoxins (described in the next section). When activated, Kupffer cells secrete a variety of cytokines, including a molecule called tumor necrosis factor alpha (TNF–α) and several types of interleukins. All of these molecules can act as inflammatory cytokines—that is, they induce an inflammatory response necessary to remove the offending toxic or foreign molecules and initiate the healing process. (For more information on cytokines and their role in alcoholic liver disease, see the article by Neuman in this issue.)

Macrophages, including Kupffer cells, and related cells appear to increase their production of cytokines in patients with alcoholic liver disease. Clinical observations have revealed that the precursor cells of macrophages (i.e., monocytes) from patients with alcohol-induced hepatitis produced greater amounts of cytokines, particularly TNF–α, than did monocytes from control patients (McClain and Cohen 1989). Moreover, patients with alcoholic liver disease had higher levels of TNF–α in their blood (Bird et al. 1990). These data have been confirmed in animals chronically exposed to alcohol (Honchel et al. 1992). Such observations suggest that TNF–α is a critical factor in alcoholic liver disease, a hypothesis that has been confirmed in animal models using rodents (Iimuro et al. 1996; Yin et al. 1999).

Results of other animal studies also have supported the role of TNF–α in alcoholic liver disease. For example, researchers have analyzed cells from a mouse strain that lacks a protein with which TNF–α interacts (i.e., a TNF–α receptor). Several TNF–α receptors exist, the primary one being TNF R1. Researchers found that mice in which TNF R1 was inactivated (i.e., TNF R1 knockout mice), thereby preventing TNF–α from exerting its effects, were resistant to alcohol-induced liver disease (Yin et al. 1999).

In addition to producing cytokines, activated Kupffer cells are the major source of reactive oxygen species (ROS) in the liver. ROS are oxygen-containing molecules that are highly reactive with other complex molecules in the cells (e.g., proteins, fat molecules [i.e., lipids], and DNA). ROS such as superoxide, hydrogen peroxide, and hydroxyl radicals have been implicated in the development of liver damage. Excessive levels of ROS within a cell and/or the lack of molecules that can eliminate ROS (i.e., antioxidants) leads to a state called oxidative stress that is detrimental to the cell. (For more information on how ROS and oxidative stress contribute to the development of alcoholic liver disease, see the article in this issue by Wu and Cedarbaum.)

One important ROS is superoxide, which in activated Kupffer cells is generated by the enzyme NADPH oxidase. Several experiments using a variety of antioxidants and inhibitors of ROS-producing enzymes have explored the relationship of superoxide generation and oxidative stress to alcohol-induced liver injury. For example, studies in which rats received chronic alcohol administration have shown that preventing superoxide production by inhibiting NADPH oxidase activity could reduce the alcohol-induced liver injury (Kono et al. 2001). Similarly, antioxidant compounds and extracts such as green tea and cocoa— which generally reduce ROS production and, consequently, oxidative stress—also provided substantial protection against alcoholic liver disease in rats (Arteel et al. 2002; McKim et al. 2002). Interestingly, in most studies using antioxidants, the alcohol-induced production of TNF–α also was reduced, suggesting that oxidative stress promotes TNF–α production.

## What Is Endotoxin?

One substance that can effectively activate Kupffer cells is bacterial endotoxin. This molecule, also known as lipopolysaccharide, is a component of the cell walls of some of the bacteria that normally inhabit the intestine. When the bacteria die, the endotoxin is released into the intestine, from which some of it can cross the intestinal wall and enter the bloodstream. Higher-than-normal amounts of endotoxin entering the bloodstream or tissues can cause fever, chills, shock, and various other symptoms and can often lead to more severe conditions, such as endotoxemia or adult respiratory distress syndrome (ARDS).

Several lines of research have shown that gut-derived endotoxin plays a critical role in alcoholic liver disease. For example, studies have found that patients with alcoholic liver disease have elevated levels of endotoxin circulating in the blood (Fukui et al. 1991). Furthermore, in experiments with rodents, researchers have found that eliminating all bacteria and, consequently, all the endotoxin from the intestine (e.g., by using antibiotics) completely prevented alcohol-induced liver injury (Adachi et al. 1995). Similarly, by lowering the number of intestinal bacteria through other means (e.g., administration of lactobacillus bacteria, which are present in yogurt), investigators could curb the rise in endotoxin levels in alcohol-treated animals (Nanji et al. 1994).

**Figure 1:**
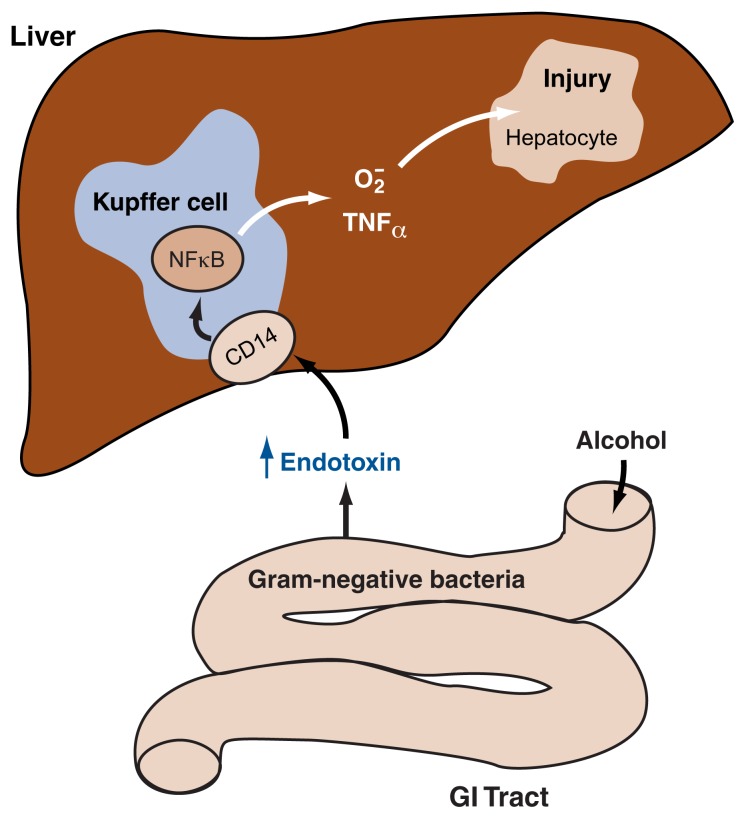
Model of the association between endotoxin release, Kupffer cell activation, and liver injury. Following chronic alcohol ingestion, endotoxin released from certain intestinal bacteria moves from the gut into the bloodstream and into the liver. There the endotoxin activates Kupffer cells—a type of immune cell (i.e., macrophages) residing in the liver—by interacting with a molecule called CD14 located on the surface of those cells. This interaction causes the production of the regulatory nuclear factor kappa B (NFκB), which in turn leads to the generation of significant amounts of cytotoxic factors, namely superoxide radicals (O_2_^•^) and various signaling molecules (i.e., cytokines), most prominently TNF–α. TNF–α has been shown to be an essential factor in the injury to primary liver cells (i.e., hepatocytes) associated with alcoholic liver disease.

As endotoxin crosses the intestinal barrier and enters the bloodstream, it interacts with the Kupffer cells in the liver, thereby activating them. Thus, it was hypothesized that Kupffer cell activation by endotoxin derived from intestinal bacteria causes those cells to generate superoxide and TNF–α, both of which can lead to tissue damage in the liver (see figure 1). Experiments on isolated cells grown in culture (i.e., in vitro experiments) as well as other experimental approaches have confirmed this hypothesis. For example, endotoxin did not induce the production of superoxide or TNF–α in isolated Kupffer cells that contained excess amounts of an antioxidant capable of eliminating superoxide (i.e., the enzyme superoxide dismutase) (Wheeler et al. 2000). This finding supports the connection between endotoxin, Kupffer cells, and superoxide production.

## Mechanisms and Consequences of Kupffer Cell Activation by Endotoxin

The idea that gut-derived endotoxin is central to the activation of Kupffer cells in alcoholic liver disease gave rise to further questions about endotoxin’s specific role in the process:

- How does endotoxin leak from the gut into the bloodstream?
- How does endotoxin activate the Kupffer cells?
- What are the consequences of Kupffer cell activation?

Answers to these questions have become clearer in recent years thanks to several research advances, especially the development of new genetic mouse models and the landmark discovery of a molecule located on the Kupffer cells that can bind to and interact with endotoxin (i.e., the LPS receptor). Eventually these emerging answers may contribute significantly to new therapies for alcoholic liver disease.

### Changes in Gut Permeability Lead to Increased Endotoxin Levels in the Blood

Several mechanisms may underlie the significant increase in endotoxin levels in the bloodstream following chronic alcohol use. According to one hypothesis, chronic alcohol use leads to higher endotoxin levels in the blood because it prevents Kupffer cells from effectively clearing these molecules from the circulation (Rivera et al. 1998). In addition, increased absorption of endotoxin from the intestine may play a role in alcohol-induced liver disease (Nolan 1975; Adachi et al. 1994). Researchers found that, in rats, acute ingestion of high alcohol concentrations facilitated the absorption of endotoxin from the animals’ small intestine by increasing intestinal permeability—that is, the degree to which the cell wall allows the passage of various molecules, including endotoxin, into the blood (Tamai et al. 2000). Other studies have shown that high alcohol concentrations can directly damage the cells lining the interior of the intestine (i.e., the intestinal epithelium), thereby impairing the ability of the epithelium to serve as a barrier preventing access of unwanted substances from the intestine to the bloodstream (Ma et al. 1999). The exact mechanism by which alcohol disrupts this protective barrier to endotoxin still is unknown, however.

Interestingly, females have higher levels of endotoxin in the blood after chronic alcohol exposure than do males (Kono et al. 2000*a*), suggesting that females may be more susceptible than males to alcohol-induced increases in gut permeability to endotoxin. These variations may be related to differences between male and female hormone systems, because researchers have demonstrated that gut permeability is significantly increased in animals treated with the female hormones estradiol and progesterone (Konno et al. 2002). Whether this mechanism explains the long-standing observation that women are more susceptible than men to alcohol-induced liver damage, however, has yet to be determined.

### How Endotoxin Activates Kupffer Cells

Researchers have identified a protein called CD14, which is located on the surface of Kupffer cells, as the receptor to which endotoxin binds (Wright et al. 1990). CD14 must be present for endotoxin to activate Kupffer cells. This activation involves the transmission of some kind of signal from the Kupffer cell’s exterior to the interior, where it triggers the numerous biochemical reactions through which an activated Kupffer cell performs its functions.

CD14 is located exclusively on the outside of each Kupffer cell and does not reach through the membrane surrounding the cells into the cell’s interior. Therefore, researchers have postulated that endotoxin also must interact with another receptor which transmits a signal into and within the Kupffer cell. Indeed, investigators recently identified several molecules called Toll-like receptors (TLR)^2^ that serve as coreceptors for endotoxin, with different TLR proteins interacting with endotoxin from different bacteria (Beutler and Rietschel 2003). The receptor most relevant to endotoxin-induced alcoholic liver disease is TLR4.^3^

**Figure 2:**
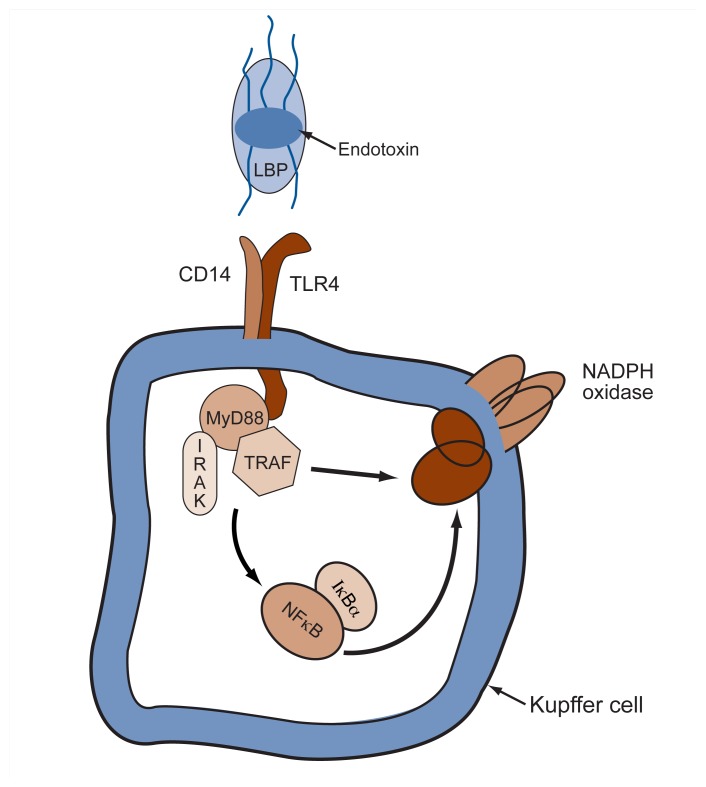
How endotoxin activates Kupffer cells in the liver. Endotoxin, also called lipopolysaccharide (LPS), in association with soluble LPS-binding protein (LBP), interacts with a receptor complex consisting of the proteins CD14 and TLR4. This interaction initiates a variety of signaling cascades in the cell. One of these cascades, which involves interleukin–1 receptor-associated kinase (IRAK) and the associated proteins MyD88 and TRAF, acts on a regulatory molecule called nuclear factor kappa B (NFκB), which is inactive in the cell if it is associated with the inhibitory molecule IκBα. In response to the signals initiated by endotoxin binding, IκBα is released from NFκB, leading to activation of NFκB. This activation, in turn, results in a number of different responses, such as the generation of superoxide through the NADPH oxidase complex and the production of cytokines. NOTE: MyD88 = myeloid differentiation factor 88; TRAF = tumor necrosis factor receptor–associated factor.

Subsequent analyses have established the relationships between endotoxin, CD14, and TLR4 (see figure 2). CD14, together with another protein called LPS-binding protein (LBP), serves as a structural receptor for endotoxin—that is, it binds to the endotoxin and physically attaches it to the Kupffer cell. The endotoxin can then interact with TLR4, and this interaction generates a signal that is transmitted by TLR4 to the cell’s interior.

Animal experiments have confirmed the importance of TLR4 in endotoxin-mediated liver damage. For nearly 25 years, researchers have known that animals from the C3H/HeJ strain of mice do not suffer adverse effects after being treated with endotoxin (Glode et al. 1976). Recent studies have shown that these mice carry a mutation in the *tlr4* gene which alters an important amino acid in the part of the Tlr4 protein that is located on the inside of the Kupffer cell (Qureshi et al. 1999; Poltorak et al. 1998). As a result, the altered protein cannot send signals into the Kupffer cells that initiate the cells’ usual response to endotoxin; thus these mice are resistant to endotoxin’s effects.

Researchers then hypothesized that if endotoxin is critical for the development of alcoholic liver disease, C3H/HeJ mice should be resistant to liver injury caused by chronic alcohol administration. Investigation of this hypothesis was made easier by two methodological advances: First, a method for administering alcohol directly into animals’ stomachs (i.e., intragastric infusion) (see the article by Nanji and French in this issue), was modified for use with mice, which are more commonly used in research. Second, using genetic engineering, investigators developed new mouse strains that carried specific foreign genes (i.e., transgenic mice) or lacked certain genes (i.e., knockout mice) in order to analyze the consequences of these alterations. Together, these advances enabled researchers to create mouse strains that either produce higher-than-normal amounts of certain components of the CD14/LBP/ Tlr4 complex or lack them, and then to study the effects of these changes when the animals were chronically exposed to alcohol.

These developments led to an explosion of studies that significantly expanded researchers’ understanding of the role of endotoxin in alcoholic liver disease. For example, investigators demonstrated that C3H/HeJ mice, which were resistant to endotoxin’s effects, showed no signs of alcohol-induced liver injury following 4 weeks of chronic alcohol feeding (Uesugi et al. 2001*a*). In contrast, mice without the *tlr4* mutation normally develop severe alcoholic hepatitis after consuming the same amount of alcohol. Other studies using animals in which the genes for CD14 were removed or which lacked LBP demonstrated that signaling processes mediated by the LPS receptor were critical to the development of liver disease associated with chronic alcohol administration (Yin et al. 2001*a*).

### Consequences of Kupffer Cell Activation by Endotoxin

As mentioned earlier, one consequence of Kupffer cell activation is the production of ROS, particularly superoxide, which in large amounts can lead to oxidative stress. Results of several studies suggest that the oxidative stress associated with chronic alcohol consumption is largely attributable to endotoxin-induced activation of Kupffer cells (Kono et al. 2000*a*; Yin et al. 2001*b*). This hypothesis expanded the current assumption that alcohol-associated oxidative stress results primarily from the degradation of alcohol in the liver by an enzyme system called cytochrome P450 2E1. (For more information on this enzyme system and its role in the production of ROS, see the article by Wu and Cedarbaum in this issue.)

Evidence for the Kupffer cell–centered hypothesis of oxidative stress comes from research with mice that lack a critical component of the NADPH oxidase complex (Jackson et al. 1995), the enzyme responsible for generating superoxide in activated Kupffer cells. After 4 weeks of chronic alcohol administration, these mice did not show signs of liver damage or an increase in the production of ROS (Kono et al. 2000*b*). This finding— combined with the previously mentioned research showing that malfunctioning or absent Kupffer cell receptors appear to protect against liver damage normally associated with alcohol consumption—establishes a link between endotoxin, Kupffer cell activation, and alcohol-induced oxidative stress.

### Endotoxin-Induced Intracellular Signaling Pathways in Kupffer Cells

As a result of endotoxin’s binding to CD14 and TLR4, Kupffer cells initiate various internal signaling processes. How endotoxin triggers these signaling pathways has been the subject of increasing research attention, given the importance of endotoxin and Kupffer cells in the development of alcohol-induced liver injury and other liver diseases.

As discussed earlier, endotoxin activates Kupffer cells through interaction with the receptor complex consisting of LBP, CD14, and TLR4. When endotoxin binds to this complex of molecules, TLR4 activates a molecule called interleukin–1 receptor-associated kinase (IRAK–1). The term “kinase” means that this enzyme can add phosphate groups to other molecules, such as enzymes, in the cells. Adding phosphate groups to various enzymes, or removing them, is important for activating or deactivating those enzymes and thereby conveying signals from the cell surface into and within all cells. For example, IRAK–1 sets off a series of signals leading to the activation of an important regulatory molecule called nuclear factor kappa B (NFκB) (Beutler and Rietschel 2003), which is crucial for regulating the activities of many genes and the cellular processes governed by those genes. Numerous other signaling pathways in Kupffer cells also are activated by endotoxin (see figure 2).

IRAK–1 is rapidly activated and then again inactivated or degraded in response to endotoxin’s activation of macrophages, including Kupffer cells (Li et al. 2000). This finding indicates both that IRAK–1 is essential for endotoxin-mediated signaling and that IRAK–1 may regulate the cellular response to endotoxin. Moreover, changes in IRAK–1 activity may serve to regulate the cascade of signals that has been triggered by endotoxin. Thus, altered IRAK–1 expression and activity may decrease signaling events mediated by TLR4 as well as the production of cytokines (i.e., a negative feedback mechanism).

When it became evident that endotoxin alters IRAK–1 activity, researchers postulated that acute alcohol exposure would lead to changes in IRAK–1 as well. Consistent with this hypothesis, Yamashina and colleagues (2000) found that in mice, alcohol administration rapidly suppressed the production and activity of IRAK–1 in the liver. In addition, IRAK–1 production was strongly correlated with the degree to which liver cells responded to endotoxin levels after acute alcohol administration.

Changes in IRAK–1 activity may be central to the phenomena of tolerance and sensitization. Tolerance means that the cells become less sensitive than normal to a stimulus (i.e., are hyporesponsive), whereas sensitization means that the cells become more sensitive than normal (i.e., are hyper-responsive). Scientists have known for some time that in addition to causing Kupffer cells to be activated by endotoxin, alcohol alters the degree to which these cells respond to endotoxin. Alcohol can cause both tolerance and sensitization to endotoxin, depending upon conditions: Acute alcohol administration typically induces tolerance, whereas chronic alcohol administration sensitizes or “primes” the liver to the toxic effects of endotoxin (Mathurin et al. 2000). Compelling evidence indicates that changes in the levels and activity of IRAK–1 and CD14 may underlie these observations (Yamashina et al. 2000; Wheeler and Thurman 2003).

As the previous sentence implies, levels of CD14 on Kupffer cells are variable rather than constant. Normally Kupffer cells have relatively few CD14 molecules on their surface (Antal-Szalmas 2000). However, numerous stimuli, including endotoxin, can lead to an increase in CD14 levels (Yamamoto et al. 1998). In humans, Kupffer cells in the normal liver have a low number of CD14 molecules, but this number increases in different inflammatory liver diseases. In rodents, CD14 levels in the liver also rise in many liver diseases, including alcoholic and cholestatic^4^ liver injury. Additional animal models have demonstrated that the levels of CD14 on macrophages vary with different stages of alcohol-induced damage (Kono et al. 2000*a*; Kishore et al. 2002). Finally, studies in mice have demonstrated that after acute alcohol exposure, the levels of CD14 mRNA—the intermediary molecule required for production of the CD14 protein from the genetic information encoded in the DNA—increase rapidly in the liver (Wheeler and Thurman 2003).

The physiological significance of these changes in the levels and activities of CD14 and IRAK–1 in Kupffer cells is not entirely clear, but these modifications could determine the liver’s sensitivity to the toxic effects of endotoxin. In addition, emerging data suggest that changes in the levels of TLR4 and other related proteins may be associated with various diseases.

## Potential Targets for the Treatment of Alcoholic Liver Disease

One of the main purposes of investigating the role of endotoxin-induced signaling events in alcoholic liver disease is to develop therapeutic approaches that can interrupt these processes, thereby preventing or at least ameliorating the resulting liver disease. One relatively unspecific approach has been to use a variety of antioxidant compounds and extracts to reduce the amount of ROS produced by activated Kupffer cells. This approach has been successful in experimental models. Most antioxidant compounds and dietary extracts, however, cannot target the production or accumulation of specific ROS. Therefore, we cannot definitively determine whether using antioxidants is effective against ROS generated by endotoxin-induced superoxide or against other mechanisms of oxidative stress associated with chronic alcohol abuse.

Another successful experimental approach has been the use of genetically engineered viruses that produce excessive amounts of (i.e., overexpress) specific enzymes and signaling molecules. For example, treating animals with a virus that overexpressed an enzyme which helps to convert superoxide into harmless products (i.e., cytosolic Cu/Zn superoxide dismutase) almost completely prevented alcohol-induced liver injury and reduced alcohol-induced TNF–α production in the animals (Wheeler et al. 2001). Similar results were obtained using a genetically engineered virus to overexpress a molecule that can suppress the activation of NFκB (Uesugi et al. 2001*b*). Whether such virus-mediated gene transfer will be of clinical use, however, has yet to be determined.

As researchers rapidly learn more about the role of TLR4 and other TLR molecules in signal transmission in macrophages, it becomes more likely that additional important therapeutic targets will be identified. For example, inhibitors that specifically interfere with signal transmission by TLR4 should be ideal candidates for potential therapy of alcoholic liver disease.

With the convincing demonstration of the role of endotoxin in pathology associated with chronic alcohol exposure and further discoveries related to TLR activity, the next few years could yield exciting advances in liver disease research. To date, there has been no effective therapy for alcoholic liver disease. Now, however, researchers and clinicians are hopeful that these approaches will result in novel and effective therapies for this debilitating disease.

## Footnotes

1 In addition to the Kupffer cells, the primary liver cells (i.e., hepatocytes) also produce and release the molecules that attract inflammation-producing cells to the liver.

2 These receptors derive their name from the fact that their structure is similar to that of a protein called Toll previously identified in fruit flies.

3 By convention, the human protein is written as TLR4, the corresponding mouse protein is written as Tlr4, and the gene encoding the mouse protein is written as *tlr4*.

4 Cholestatic liver disease is a type of liver disease (i.e., hepatitis) that leads to an interruption of the flow of bile from the liver to parts of the intestine.
